# Anti-angiogenesis and Immunotherapy: Novel Paradigms to Envision Tailored Approaches in Renal Cell-Carcinoma

**DOI:** 10.3390/jcm9051594

**Published:** 2020-05-24

**Authors:** Antonella Argentiero, Antonio Giovanni Solimando, Markus Krebs, Patrizia Leone, Nicola Susca, Oronzo Brunetti, Vito Racanelli, Angelo Vacca, Nicola Silvestris

**Affiliations:** 1 Medical Oncology Unit, IRCCS Istituto Tumori “Giovanni Paolo II” of Bari, 70124 Bari, Italy; argentieroantonella@gmail.com (A.A.); antonio.solimando@uniba.it (A.G.S.); dr.oronzo.brunetti@tiscali.it (O.B.); 2 Department of Biomedical Sciences and Human Oncology, Section of Internal Medicine ‘G. Baccelli’, University of Bari Medical School, 70124 Bari, Italy; patrizia.leone@uniba.it (P.L.); susnic2@gmail.com (N.S.); vito.racanelli1@uniba.it (V.R.); angelo.vacca@uniba.it (A.V.); 3 Department of Urology and Pediatric Urology, University Hospital Würzburg, 97080 Würzburg, Germany; Krebs_M@ukw.de; 4 Comprehensive Cancer Center Mainfranken, University Hospital Würzburg, 97080 Würzburg, Germany; 5 Department of Biomedical Sciences and Human Oncology, University of Bari Medical School, 70124 Bari, Italy

**Keywords:** renal cell carcinoma, angiogenesis, immune-checkpoint inhibitor, tumor microenvironment, molecular subtypes, prognostic-biomarkers, predictive factors

## Abstract

Although decision making strategy based on clinico-histopathological criteria is well established, renal cell carcinoma (RCC) represents a spectrum of biological ecosystems characterized by distinct genetic and molecular alterations, diverse clinical courses and potential specific therapeutic vulnerabilities. Given the plethora of drugs available, the subtype-tailored treatment to RCC subtype holds the potential to improve patient outcome, shrinking treatment-related morbidity and cost. The emerging knowledge of the molecular taxonomy of RCC is evolving, whilst the antiangiogenic and immunotherapy landscape maintains and reinforces their potential. Although several prognostic factors of survival in patients with RCC have been described, no reliable predictive biomarkers of treatment individual sensitivity or resistance have been identified. In this review, we summarize the available evidence able to prompt more precise and individualized patient selection in well-designed clinical trials, covering the unmet need of medical choices in the era of next-generation anti-angiogenesis and immunotherapy.

## 1. Introduction 

Angiogenesis inhibition remains one of the most active approaches in the treatment of advanced kidney tumors. Although tumor heterogeneity can be a therapeutic obstacle [1] angiogenesis-related mechanisms represent a truncal event in renal cell carcinoma (RCC) biology, particularly in clear-cell histotype. Indeed, the alterations of the HIF/VEGF axis are deemed to be the fundamental target [2], even aiming at overcoming drug resistance [3]. This evidence explains the clinical success of sequential strategies employing tyrosine kinase inhibitors (TKI) [4,5,6]. Nonetheless, recent evidence warrants taking into consideration a more complex biological scenario accounting for RCC pro-angiogenetic mechanisms. However, the RCC boosted neo-vessel formation does not behave as an oncogene addiction that characterizes other malignancies [7]. Indeed, a complex architecture accounts for the RCC heterogeneity, coexisting with a tumor microenvironment educated as a tolerogenic niche [8]. This sophisticated milieu prompts us to uncover immunotherapy to be an effective up-front treatment option.

Nevertheless, not all patients seem to benefit equally from immune-checkpoint inhibition, being characterized by either primary- or secondary-refractoriness [9,10,11,12]. Indeed, the subset of individuals classified as favorable risk seems to be an oasis in which the TKI sequence followed by TKI may still represent a logical choice [13,14,15]. Conversely, despite ambitious attempts aimed at dissecting the biology behind RCC [16,17], the criteria used to stratify patients’ risk and response predictions remain largely elusive, since the evidence on which we currently base the hypothesis-generating indications have been adapted from clinical and laboratory criteria. Peculiar subgroups treated by single agents inhibiting angiogenesis, even in a stepwise fashion [13], hold great potential in terms of disease control and long survival. Indeed, molecular signatures exist and may perhaps identify angiogenesis-driven tumors, able to translate the plethora of already broadly corroborated evidence obtained from in vitro [18,19], in embryo [20,21,22] and in vivo assays [23,24]. Contrariwise, specific subjects can be considered non-angiogenesis addicted. In these cases, combination immunotherapy or less selective TKI may constitute a more efficient upfront strategy [25,26]. From this perspective, the phenotypic deconvolution aiming at biomarkers identification and response prediction, can support customizing RCC treatment. From this standpoint, it is tempting to propose a combination of anti-angiogenic and immune-checkpoint inhibitors (ICI), especially when driven by compelling molecular signatures [27,28].

## 2. Historical Evolution/Perspective of Prognostic Systems in mRCC

The prognosis of patients with renal cell carcinoma (RCC) is influenced by the anatomical, histological, clinical and molecular characteristics of the neoplasm. The use of anatomo-histological prognostic factors is further supported by higher levels of evidence compared to clinical and molecular factors. Anatomical features are described in clinical practice through the TNM classification system. Anatomical classification systems such as the PADUA (Preoperative Aspects and Dimensions Used for an Anatomical classification system), the R.E.N.A.L. (Radium, Exophytic/endophytic properties, Nearness of the tumor to the collecting system or sinus, Anterior/posterior, Location relative to the polar line) and the C-index have been proposed to standardize the description of kidney tumors [29,30,31]. These classification criteria take into consideration features such as size, endo/exophytic growth, relationships with the renal hilum and collector ducts and the anterior or posterior position of the tumor. These systems are useful for assessing the potential morbidity of surgery and ablation techniques. Furthermore, in the case of metastatic neoplasia (mRCC), the prognosis is further influenced by the number and location of the metastatic sites [32,33]. The main histological features of renal carcinoma potentially holding a prognostic value are represented by the histotype (clear-cells: 70–80% of cases; papillary: 10–15%; chromophobe: 5%), grading, the presence of tumor necrosis, microvascular invasion, sarcomatoid component, and involvement of the collector system. Grading remains the most important accredited prognostic factor [34]. The WHO/ISUP classification published in 2013 proposes the replacement of the Fuhrman grade with a ISUP/WHO system ranging from I to IV, describing nucleolar characteristics, taking into account the presence of a rhabdoid component in grade IV and/or the presence of the sarcomatoid variant. So far, this classification has been validated for clear-cell and papillary tumors so far. Among the other histotypes nuclear grading it holds a descriptive role [35], with scanty translational consequences. Moreover, statistical validation by univariate analysis corroborated the prognostic impact of the tumor histotype, while describing the clear-cell carcinoma as the most aggressive subtype, followed by the papillary and chromophobe. Conversely, in multivariate models, the prognostic significance of the histotype was deemed not significant, suggesting that the stage of disease and tumor grading harbor a greater impact on the prognosis than the histotypic characteristic per se. Furthermore, the papillary carcinomas can be further subdivided into two subtypes with different clinical outcomes: type I, low grade tumor with favorable prognosis, and type II, high grade tumor with increased dissemination potential [36,37,38]. In a retrospective multivariate analysis of over 600 patients suffering from metastatic renal carcinoma and enrolled in clinical trials in the 1980s, Elson et al. identified five survival indicators: ECOG PS, the time period between diagnosis and first systemic treatment, the number of metastatic sites, previous systemic therapies and weight loss. Based on these factors, the authors stratified patients into five groups characterized by different survival [35]. Subsequently, numerous integrated models were outlined aimed at analyzing clinical, pathological factors and laboratory parameters in order to predict survival and identify patients with a high risk of relapse. Among these, the two most widely used in clinical practice and experimentation are the prognostic system of the MSKCC (Memorial Sloan Kettering Cancer Center or Motzer model) and the prognostic system of the IMDC (International Metastatic RCC Database Consortium or Heng’s model) [39].

**Figure 1:**
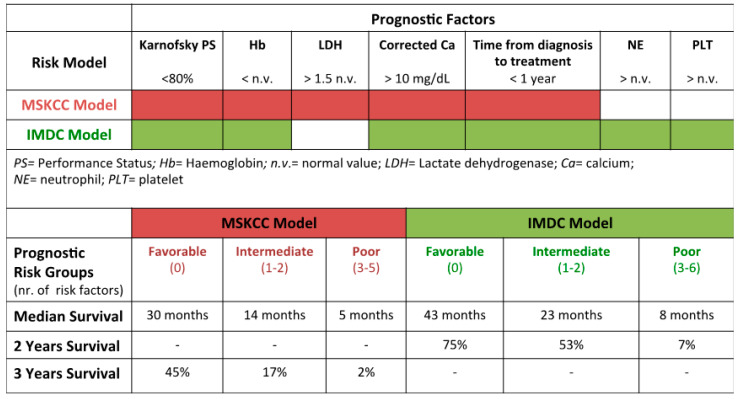
MSKCC Model (Motzer et al.) and International Metastatic RCC Database Consortium (IMDC) Model (Heng et al.): risk categories and relative median survivals in renal cell carcinoma [13,40,41,42]. The color code represents the presence of a given prognostic factors for each model: PS, Hb, LDH, corrected Ca, time from diagnosis to treatment (red) for MSKCC model; PS, Hb, corrected Ca, time from diagnosis to treatment, NE, PLT (green) for IMDC model.

In order to overcome the statistical power limitation, both in terms of sample size and number of series included in the multivariate analyses available [40], Motzer et al., in a series of 670 patients with advanced RCC and treated with immunotherapy or chemotherapy, identified five pre-treatment factors significantly related to a unfavorable prognosis, namely decreased Karnofsky PS (<80%), s high value of LDH (>1.5 times over the boundaries) and calcemia (>10 mg/dl), decreased hemoglobin concentration, and failure to perform the surgical procedure [40]. Using these variables, they stratified the patients into three groups (favorable, intermediate and unfavorable risk group) with dismal clinical outcome for the high risk subgroup; survival ranged from 20 months for the group with a favorable prognosis to 4 months for the group with a poor prognosis [40]. Next, a similar analysis was applied to 400 patients treated in the first line with IFN-α; this restriction of inclusion criteria has minimized the heterogeneity determined by previous treatments. The prognostic stratification criteria were unmodified, except for the substitution of the factor “no nephrectomy”, with the factor “time period elapsed between the diagnosis and the immunological treatment less than one year” [41] (Figure 1).

Subsequently, Heng et al., in a series of 645 patients with advanced renal cell carcinoma, identified six prognostic factors significantly related to a worse prognosis (IMDC, or Heng model). This system derives from a retrospective analysis conducted on patients with metastatic renal cell carcinoma treated with sunitinib, sorafenib or bevacizumab + interferon alfa-2a. Patients who had received a first line of treatment with cytokines and VEGF/VEGFR inhibitors as second-line treatment were also included in the analysis. Six prognostic factors have been identified: Karnofsky PS, low hemoglobin level, high corrected serum calcium, period from diagnosis to treatment ˂ 1-year, high absolute neutrophil count, and high platelet count. Subjects were divided into different subgroups according to clinical risk: favorable (*n* = 157), for whom the median overall survival (OS) was 43.2 months and the 2-year OS was 75%; intermediate (*n* = 440), characterized by a median OS was 22.5 months and the 2-year OS was 53%; poor risk (*n* = 252) in which the median OS was 7.8 months and the 2-year OS was 7% [13,42] (Figure 1). 

## 3. New Insights in Prognostic and Predictive Biomarkers Stratification 

### 3.1. From the Cytogenetics to the Mutational Landscape of RCC

Despite the considerable efforts made to stratify patients from a prognostic standpoint by using clinical criteria, efficient prognosticators for characterization represent an unmet medical need, especially when considering the plethora of new immunomodulatory and anti-angiogenic drugs available to date. Cytogenetics pioneered the molecular investigation of patient stratification, based on Xp11.2 translocation and deletion or chromosomal aberration on 3p and 14 in RCC-impacted clinical outcomes [43,44,45]; the incidence of Xp11.2 translocation is very low, but should be searched for systematically in young patients [46]. Chromosome 3 harbors several putative oncogenes and oncosuppressors, the biological relevance of which is highlighted by von Hippel-Lindau(VHL)/HIF-1α axis, PBRM1, BAP1, SETD2 prognostic role [16,45,47,48,49]. Furthermore, numerous chromosome alterations in terms of chromosome gain or loss (i.e., gain of 7q, loss of 9p, 9q and 14q) have been highlighted and associated with worse survival (*p* < 0.001), with a prognostic but not predictive role [50].

Next, several novel biomarkers are currently being evaluated to assess the prognostic and predictive value for different response of renal malignancies treated with antiangiogenic-TKI and immunotherapy. Molecular markers can be classified according to their physiological location into tissue and soluble factors [51]. Among the above-mentioned traditional histological features, carbonic anhydrase IX (CaIX) [52], CXCR4 [53,54], HIF-1α and HIF-2α [55] have been reported to predict response to sorafenib or sunitinib as well as improved progression-free survival (PFS), despite no consistent impact on OS being reported. Specifically, tumor shrinkage gained by sorafenib treatment significantly differed between CaIX^high^ vs. CaIX^low^ cases (−13% vs. +9%) [52]. Moreover, D’alterio et al. and Guo et al. independently revealed CXCR4 expression level to be significantly correlated to sunitinib response and improved PFS in patients treated with sorafenib, respectively (median PFS 20 vs. 6 months, in CXCR4^low/high^, *p* = 0.038) [53,54]. Furthermore, patients’ stratification—according to HIF-1α expression level—was also able to predict improved PFS in the HIF-1α^high^ over the HIF-1α^low^ sunitinib-treated-subgroup (42.0 weeks vs. 30.4, respectively, *p* = 0.034) [56].

Furthermore, PD-L1 expression in tumor cells and in tumor-infiltrating immune cells is associated with poor clinical outcome (cancer-specific death, *p* < 0.05) [57], without a predictive role of response to cabozantinib and axitinib plus anti-PD1/PD-L1 [4,58,59,60,61]. Nevertheless, available data are still debated, since interesting results showed a clinical value in predicting response to ipilimumab combined with nivolumab treatment [9].

The assessment of the soluble factors evaluation has also been extensively investigated in the prognostic stratification attempts, uncovering VEGF/VEGFR, LDH, IL-6, IL-8, osteopontin (OPN), HGF and TIMP1 to be significant drivers of a patient’s prognosis and response to therapy [62,63,64,65]. High serum VEGF levels reflected an aggressive tumor biology and kept an independent prognostic value in a multivariate analyses including MSKCC score and ECOG PS, while being able to predict a better clinical outcome over the unstratified population (*p* = 0.015) [66]. Low baseline levels of sVEGFR3 and VEGF-C were also predictive of improved PFS upon sunitinib treatment. (median PSF 36.7 weeks and 19.4 weeks in sVEGFR 3^low/high^, respectively; moreover, the median PSF was 46.1 weeks and 21.9 weeks in VEGF-C^low/high^ patients, respectively [62]. Next, IL-6, osteopontin, and TIMP-1 were integrated in a prognostic model including selected clinical variables and showing higher prognostic accuracy than IMDC model (concordance-index 0.75 vs. 0.67, respectively) [65]. Ancillary, emerging evidences uncovered nucleotide polymorphisms (SNPs) of IL-8, HIF-1α and VEGF axes to significantly impact the therapeutic outcome in RCC [67,68] as in several TKI sensitive tumors [69,70,71]; however, no validation has been achieved in statistically powered clinical studies [55,72]. 

A recent report highlighted the emerging role played by non-coding RNA, such as miRNA in RCC; in the frame of this thinking, clinically and prognostically relevant RCC subgroups were reflected by distinctive miR expression levels [73,74,75,76]. For example, Heinzelmann and colleagues identified a signature, including miR-451, miR-221 and miR-26a, which separated between metastatic and non-metastatic clear cell RCC [77]. Functionally, miRs orchestrate crucial steps in immunosurveillance and modulate cancer immune checkpoints by influencing cells of the immune system and tumor cells [78,79]. In RCC, miRs were shown to regulate HLA-G [80] and PD-L1 expression [81]. Additionally, there is a growing body of literature highlighting the prominent role of miRs in angiogenesis-related signaling [76,82,83,84]. For instance, miR-195 and miR-221 regulate the expression of VEGFR2 in various tissues, including RCC [85,86,87,88,89]. Accordingly, miR-221 expression was part of signatures predicting the response of RCC patients towards TKI/anti-angiogenic therapy in two independent studies [87,90].

Evidence from tissue and circulating pro-angiogenic factors matches with familial VHL syndrome disease-phenotype: hypoxia-inducible factors overactivation constitutes a fundamental proof of principle in hereditary clear-cell RCC (ccRCC), but also elicited comprehensive genomic characterization of sporadic tumors, by focusing on pro-angiogenic mechanisms. In ccRCC, decreased VHL activity correlated with enhanced HIF-1α expression, as well as with the consequent hyperactivation of VEGF, PDGF, TGF-α, thus leading to increased PI3-K/PKB/mTOR signaling, and tumor progression [91,92,93]. Undoubtably, the biological knowledge related to VHL pathway-driven investigation inspired novel therapeutic windows [94,95]. However, several data and meta-analyses revealed that VHL gene alteration holds neither prognostic, nor predictive value in subjects suffering from ccRCC [91,96]. 

The dismal impact on clinical outcome exerted by VHL per se can likely also be explained by the complex genomic architecture driving the malignant phenotype of RCC. Indeed, several additional genetic alterations were also frequent in ccRCC, such as somatic mutation of chromatin remodeling genes including PBRM1, SETD2 and BAP1 (38%, 13% and 11% of cases, respectively), mutation of PI3K–AKT–mTOR pathway genes (occurring in 16% of patients) comprising PTEN, MTOR and PIK3CA, loss of CDKN2A, and mutation of TP53 (16.2% and 2.6%, of subjects, respectively) [16]. CDKN2A loss, BAP-1 and TP53 mutation are associated with poorer survival in ccRCC. The poor prognostic role of CDKN2A loss has also been confirmed in papillary and chromophobe RCC histological subtypes [16]. Conversely, PBRM1 loss-of-function mutations correlated with less aggressive behavior and with better PFS and OS in advanced patients [97,98,99]. In a retrospective study, Kapur et al. revealed PBRM1 to be significantly predictive for improved median OS (10.6 vs. 4.6 years) when compared to BAP1 mutational status. Consistently, data from TCGA confirmed the UTSW cohort by showing median OS of 5.4 and 1.9 years for PBRM1 vs. BAP1 mutated cases, respectively [98]. Next, genomic annotation-model based uncovered the independent prognostic value harbored by any TP53, BAP1 and PBRM1 mutation to be relevant in improving the MSKCC model in patients treated with first-line TKI [100]. Likewise, the IMmotion150 trial, which compared, in a three-arm fashion, sunitinib over atezolizumab monotherapy and atezolizumab plus bevacizumab in treatment-naive RCC, revealed PBRM1 mutations to be correlated with improved survival in the sunitinib arm. Additionally, the ICI response prediction to anti-PD1 identified by PBRM1 mutational status apparently parallels the behavior reported in TKI-treated patients [97], warranting further statistically powered trials aimed to clarify the predictive value of PBRM1 [25].

### 3.2. Molecular Classification

Gene expression profile parallels genetic and genomic alterations and impacts the clinical outcome. The mRNA expression patterns differ among major histological subtypes as well as among each RCC subtype. Proteomics-based subtyping of ccRCC, either according to Brannon et al. (two clusters, ccA and ccB) [101], Chen et al. (three clusters, CC–e.1, CC–e.2, CC–e.3) [102], or KIRC analysis (four clusters m1–m4) [103] consistently deconvolute the biologic taxonomy of disease phenotype. Moreover, the combination of the singular subtypes can dissect three different clinical behaviors: (1) good prognosis group (cluster ccA, CC–e.2, and m1), involved chromatin modifier genes mutations, such as PBRM1; (2) poor prognosis group (cluster ccB, CC–e.3, m3), associated with higher expression of CDKN2A and hypoxia-related genes, chromatin remodeling genes mutation including SETD2 or BAP1, PI3K/AKT/mTOR pathway genes mutations, epithelial–mesenchymal transition, hypermethylation, and a metabolic shift with higher glutathione and dipeptide levels; (3) intermediate prognosis group (cluster 3, CC–e.1, m2, and m4) associated with BAP1 mutations and base-excision repair [55]. Additionally, data obtained from 942 surgical series pinpoint a molecular signature consisting of 16 genes that could predict post-surgery relapse and could be translated into clinical trials [104]. Unsupervised hierarchical cluster analysis identified different biological pathways, including vascular, cell growth or division, immune response, and inflammation phenotypes. In line with previous data, vascular and immune response phenotypes were associated with a better outcome, whereas higher expression of proliferation and differentiation genes and markers associated with inflammatory responses were associated with a worse outcome [104]. Overall, it is worth highlighting that all the above-mentioned data were generated by analyzing prognostic implications obtained from non-metastatic settings. Conversely, Beuselinck et al. performed a multi-omics analysis and identified four molecular tumor subtypes able to predict clinical outcome and response to sunitinib in metastatic ccRCC: ccrcc1 (“c-myc-up”) and ccrcc4 (“c-myc-up and immune-up”) characterized by the upregulation of MYC targets and shorter PFS, OS and poorer response to sunitinib; ccrcc2 (“classical”) and ccrcc3 (“normal-like”) with a higher expression of the pro-angiogenic HIF-VEGF-VEGFR-pathway, longer OS and better TKI response. Characteristically, the ccrcc4 subtype had a strong inflammation, BAP1 mutation, sarcomatoid dedifferentiation and decreased angiogenesis dependency, and significantly poor survival and response to sunitinib and pazopanib [105,106]. The four molecular subtypes could explain the different outcome in the IMDC risk group. The IMDC good risk group was enriched for the ccrcc2 subtype and higher angioscore; conversely, the IMDC poor risk group was enriched for the ccrcc4 subtype and lower angioscore. Nevertheless, no correlation was found in the immune score across IMDC risk groups [107]. Given that the existence of an angiogenesis-addicted, and immune-inflamed phenotypes seems to correlate with the presence of peculiar genomic signatures [108], it is tempting to speculate an ancillary role played by specific mutated genes. Remarkably, PBRM1 mutational *status* and boosted angiogenesis in ccrcc2–3 seem to have more interactions among themselves than would be expected for a random set of molecular interactions [26,108]. The different clinical outcome obtained in sunitinib-treated patients compared to avelumab alone or in combination with bevacizumab remains to be fully elucidated, and might be explained by an underlying angiogenesis-driven mechanism in this subgroup over PBRM1 wild type phenotype [25].

In a comprehensive interrogation of available datasets carried out by Hakimi et al., four clusters were also identified, shedding more light on the peculiar features of the tumor microenvironment (TME) and substantially extending the insights regarding the role of angiogenesis signatures in predicting TKI response. Specifically, this analysis highlighted the role of macrophages fingerprint within the TME and uncovered a putative angiogenesis^high^ macrophages^low^ signature to be one fundamental determinant predicting prognosis and, likely, impacting response to TKI [26]. This piece of evidence might support clinical decision while selecting approaches based on mono- vs. combination-therapy and anti-angiogenesis vs. ICI-inhibitors based approaches, also pinpointing the unexplored efficacy of CSFR1-targeting [26]. These data need to be confirmed in appropriately designed studies to be translated into clinical practice.

Collectively, the complex taxonomy behind RCC recapitulates evidences already validated in several solid [109,110] and haematological [111,112,113,114] malignancies, from the emerging role of the tumor microenvironment standpoint [115,116]: in patient clinical outcome prediction, inspired non-invasive evaluation aimed to picture the impact of cancer associated bystanders, such as circulating and cancer-associated stromal cells [117], like fibroblast [118] and endothelial cells (EC) [119]. This phenotype mirrors the behavior of several angiogenesis-addicted cancers [120,121,122,123,124], in which laboratory and angiogenesis-markers [63,125] related to the VHL [91] and mTOR (mammalian target of rapamycin) pathway [92] are also shared.

## 4. Therapeutic Window Driven by Angiogenesis and the Immune System Targeting Current Challenges

**Figure 2:**
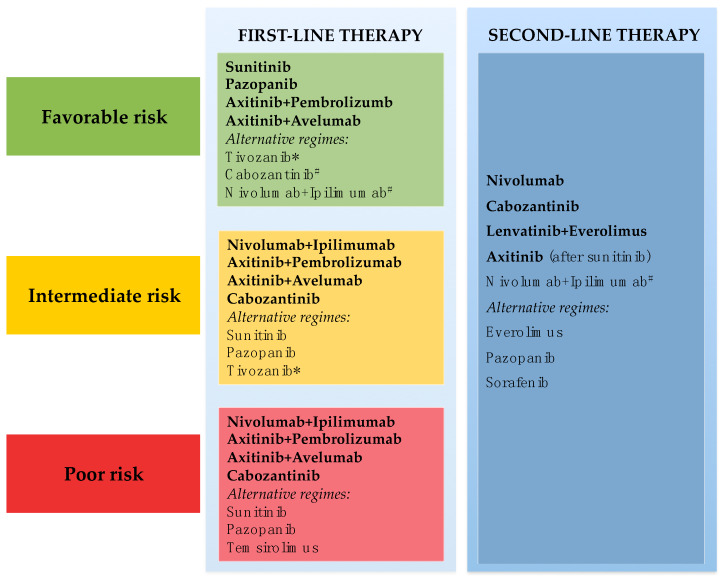
Systemic treatment of clear-cell renal cell carcinoma according to IMDC prognostic system. * Only EMA approval, ^#^ Only FDA approval.

The treatment scenario of mRCC has largely evolved in recent years, translating into an outcome improvement achieved by targeting VEGF/VEGFR pathways (bevacizumab, sorafenib, sunitinib, pazopanib, axitinib, cabozantinib and lenvatinib) [66,126,127,128,129,130,131], mTOR signaling (everolimus and temsirolimus) [132,133] and immunocheckpoint inhibitors comprising anti PD1/PD-L1 (nivolumab, pembrolizumab, avelumab, atezolizumab) [11,59,60,61] and anti-CTLA4 (ipilimumab) [134] alone or in combination therapies (Figure 2).

Due to the dynamic plethora of therapeutic options available to date, it is critical to identify criteria driving personalized approaches. Indeed, real-life clinical practice faces the significant challenge of patient selection by tailoring a TKI- vs. ICI-based and mono- vs. combination-therapeutic strategy [55,135]. Currently, besides the obvious impact of clinical individual risk profiling and comorbidities potentially influencing the safety issues, the single decision-making tool is represented by the risk stratification considered by the regulatory agencies.

Intermediate and high-risk might warrant a cabozantinib-containing regimen according to the CABOSUN study [4] that evaluated only this setting of patients. Cabozantinib, as a small molecule halting several tyrosine kinase receptors such as VEGFR-2, MET and AXL, as well as other potentially relevant kinases including RET, KIT, and FLT3, has been evaluated in the CABOSUN phase II multicenter study. In total, 157 subjects with intermediate/high risk stratified by Heng profiling were randomized to receive cabozantinib or sunitinib [4,136,137]. The CABOSUN trial met the primary end-point, showing improved PFS in the experimental arm (median PFS 8.2 months vs. 5.6 months with cabozantinib over sunitinib, respectively, supporting cabozantinib as a potential first-line treatment option for patients with advanced ccRCC of intermediate or poor risk [138]. Cabozantinib has been uncovered to also be effective in metastatic non-clear RCC in a retrospective cohort study investigating naïve and refractory cancers and all IMDC model risk groups. The median PFS was 7.0 months, and median OS was 12.0 months [139].

The combination study of nivolumab with ipilimumab (CheckMate-214 study) including all-comers showed an ICI benefit in the intermediate/high-risk population only, apparently with a detrimental effect in low-risk patients, where sunitinib conferred an improved clinical outcome [9,134]. The phase 3 trial included 847 patients with untreated advanced RCC who were randomly assigned to receive either nivolumab in combination with ipilimumab, or sunitinib. In the latest update, presented at 2020 Genitourinary Cancers Symposium at median 42 months of follow-up, the combination immunotherapy continued to be associated with improved OS and PFS compared to sunitinib arm (median 47.0 vs. 26.6, and 12 vs. 8.3 months, respectively, and 42-month rates of 52% versus 39%, and 35% versus 19%, respectively). PFS curves plateaued after 30 months at around 35% with nivolumab plus ipilimumab. An exploratory efficacy analysis restricted to the 249 favorable-risk participants established sunitinib to be more active when compared to ICI in this patient subset, gaining a median PFS of 27.7 vs. 17.8 months and ORRs of 54% vs. 29% [140].

However, the clinical and pathological features not entirely mirroring the complex biology of the tumor should be adapted to the novel agent’s era. Specifically, Heng criteria [13] and prognostic factors were developed to inform patients about their prognosis and in order to compare the results of different trials [141]. Conversely, such stratification tools are not expected to perform efficiently in therapeutic strategy selection. The Checkmate 214 study represented a paradigm shift, with the potential to picture and weigh the single prognostic factors quantity over the global additive effect on the clinical outcome [9,134]. Moreover, the platelet count and the calcium levels had a more significant impact than was usually observed [142].

The recently published data regarding the combination of anti-angiogenic and anti-PD1 treatment (axitinib in combination with pembrolizumab [59] or avelumab [60]) compared to sunitinib demonstrated a benefit from the combination across the population, regardless of risk class and PD-L1 expression.

In the phase III KEYNOTE-426 study, the majority of patients displayed intermediate or poor risk disease as assessed by IMDC criteria and sarcomatoid features in 18% of the patients. PFS was 15.1 and 11.1 months in the pembrolizumab/axitinib and in the sunitinib group, respectively. Pembrolizumab plus axitinib demonstrated effectiveness and good safety for patients with clear cell mRCC, with an impressive 59% objective response rate.

The Javelin renal 101 study dichotomized patients into two classes—PD-L1^positive/negative^—choosing immunohistochemistry expression as class boundary and by declaring as co-primary outcome OS and PFS assessment in PD-L1^positive^. Avelumab/axitinib performed better than sunitinib in terms of both PFS and ORR, regardless of PD-L1 expression [60]. Conversely, a trend of enhanced efficacy within the PD-L1^positive^ subgroup was observed in the atezolizumab plus bevacizumab arm compared to sunitinib in the IMmotion151 trial (median PFS 11.2 vs. 7.7 months, respectively; *p* = 0.0217) [61]. Preliminary results of a new combination of TKI (cabozantinib) plus anti-PD1 (nivolumab) promise a clinically meaningful benefit (NCT03141177) and warrants further investigation regarding the chance of anti-angiogenic strategies combined to ICI. Additional information aiming to clarify whether this approach might benefit as pure synergic strategy or by intercepting a broader disease spectrum irrespective of patients’ selection remains as-of-yet unknown.

Collectively, evidence generated by the above-mentioned trials uncovered neither the risk class nor the PD-L1 expression as being efficient in predicting the response to therapy. Thus, several omics attempts retrospectively analyzed the available data. Nonetheless, as a first in class prospective study, the IMmotion150 phase II emphasized the translational role of TME deconvolution at the transcriptomic level, suggesting that the outcome prediction with anti-angiogenic drugs and ICI is applicable upfront in mRCC [25]. In detail, a gene expression profiling fingerprint has been proposed according to different phenotypes, clustered using expression ranks boundaries of pro-angiogenic, pre-existing immune- and myeloid tolerogenic-associated molecular subgroups [25]. Consequently, as expected, angiogenic blocking by sunitinib was highly active in Angiogenesis^High^ patients, whilst atezolizumab alone seems to halt tumor activity in immunogenic cancers and dismal myeloid inflammation (Teff^High^ Myeloid^Low^). Regarding the combination of ICI plus sunitinib, although the authors comprehensively demonstrated a direct impact of immune- and inflamed-infiltration (Teff^High^ Myeloid^High^) [25], it is still debated whether combining anti-angiogenic and immunological checkpoint inhibitors without proper selection, more than what would be necessary, constitutes a synergistic strategy [143]. Nonetheless, robust and compelling preclinical [28,143] and clinical [26] evidence supports the biological ecosystem dissection as the future driver of patient selection for choosing candidates among ICI/anti-angiogenic strategies: different biological RCC behaviors pinpoints the tight correlation existing by intermediate/high risk profile, tumor angiogenesis and indirect immune-tolerogenic milieu. The roadblocks in standardizing biomarkers in clinics are due to the lack of data able to deconvolute RCC biological characteristics derived from prospective studies. Moreover, additional caveats restraining the real-life translation of the biological RCC taxonomy are constituted by the patient population heterogeneity and by the absence of a clinical stratification model accounting for next-generation immune-targeted therapy. Statistically powered clinical studies are expected to be carried out, aimed at further validating the promising pioneering results [144]. State-of-the art molecular dissection of RCC subtypes should guide clinical trials’ designs, in order to efficiently tailor the best therapeutic option upfront. An Achilles’ heel of the modern approach proposed might be the applicability of changing clinical tools; however, the rational and efficient use of the novel agents available would also prevent the inevitable financial toxicity of the integrated stepwise RCC management. These data will be more than a determinant in a dynamic evolving sequential treatment strategy, thereby deeply impacting further therapy. Details from the most recent clinical trials are summarized in Table 1.

## 5. Conclusions

We are currently entering the third era of mRCC therapy with the challenging aim of combining immune–immune and immune–VEGFR-TKI, which is a direct portrait of the peculiar underlying pathophysiology of disease, being dependent on angiogenesis and the close connection between cancer cells and the immune system. The lack of direct comparisons, as well as different study designs and patient stratification, considered as major limits could also represent a caveat in order to better tailor clinical decisions. Nonetheless, though prognostication is mandatory, biological correlates are highly needed. Using immunotherapy, it is mandatory to design clinical trials with a robust immunological background.

## Figures and Tables

**Table 1:** Phase II/III trials of novel therapeutic approaches vs. Sunitinib for untreated patients with metastatic renal cell carcinoma. PFS= progression free survival; OS = overall survival; ORR = overall response rate; CR = complete response; AE = adverse events; NA = not available; NR = not reached [59,60,61,136,140].

	Cabozantinib (*n* = 79) vs. Sunitinib (*n* = 78) CABOSUN	Nivolumab + Ipilimumab (*n* = 550) vs. Sunitinib (*n* = 546) CheckMate 214	Pembrolizumab + Axitinib (*n* = 432) vs. Sunitinib (*n* = 429) KEYNOTE 426	Avelumab + Axitinib (*n* = 442) vs. Sunitinib (*n* = 444) JAVELIN Renal 101	Atezolizumab + Bevacizumab (*n* = 454) vs. Sunitinib (*n* = 461) IMmotion 151
IMDC Score					
Favorable	−	23%	32%	21%	20%
Intermediate	81%	61%	55%	61%	69%
Poor	19%	17%	13%	16%	12%
PD-L1 expression ≥ 1%	23%	24%	60.5%	63.2%	40%
Primary end-point	PFS	OS, PFS, ORR (intermediate + poor risk)	OS, PFS (ITT)	PFS, OS (PD-L1+)	PFS (PD-L1+), OS (ITT)
Secondary end-point	OS, ORR	OS, PFS, ORR (ITT)	ORR	PFS, OS (ITT), ORR	PFS (ITT), OS (PD-L1+), ORR
Median follow-up (months)	34.5 months	42.0 months	12.8 months	9.9 months (Av. + Ax.) 8.4 months (Sun.)	15.0 months for PFS 24.0 months for OS
Median PFS (months)					
Experimental arm vs. Sunitinib (ITT)	8.6 vs. 5.3 months	12.5 vs. 12.3 months	15.1 vs. 11.1 months	13.8 vs. 8.4 months	11.2 vs. 8.4 months
Experimental arm vs. Sunitinib (other population)	*NA*	12.0 vs. 8.3 months (intermediate + poor risk)	15.3 vs. 8.9 months (PD-L1+)	13.8 vs. 7.2 months (PD-L1+)	11.2 vs. 7.7 months (PD-L1+)
Median OS (months)					
Experimental arm vs. Sunitinib (ITT)	26.6 vs. 21.2 months	*NR* vs. 38.4 months	*NR*	*NR*	33.6 vs. 34.9 months
Experimental arm vs. Sunitinib (other population)	*NA*	47.0 vs. 26.6 months (intermediate + poor risk)	*NA*	*NR*	34.0 vs. 32.7 months (PD-L1+)
ORR (%)					
Experimental arm vs. Sunitinib (ITT)	20% vs. 9%	39% vs. 33%	59.3% vs. 35.7%	51.4% vs. 25.7%	37% vs. 33%
Experimental arm vs. Sunitinib (other population)	*NA*	42% vs. 26% (intermediate + poor risk)	*NA*	55.2% vs. 25.5% (PD-L1+)	43% vs. 35% (PD-L1+)
CR (%)					
Experimental arm vs. Sunitinib (ITT)	0.8% vs. 0%	11% vs. 2%	5.8% vs. 1.9%	3.4% vs. 1.8%	5% vs. 2%
Experimental arm vs. Sunitinib (other population)	*NA*	10% vs. 1% (intermediate + poor risk)	*NA*	4.4% vs. 2.1 (PD-L1+)	9% vs. 4% (PD-L1+)
Grade ≥ 3 AEs					
Experimental arm vs. Sunitinib	68% vs. 65%	46% vs. 63%	62.9% vs. 58.1%	71.2% vs. 71.5%	40% vs. 54%
